# Botulinum Neurotoxins and Botulism: A Novel Therapeutic Approach 

**DOI:** 10.3390/toxins3050469

**Published:** 2011-05-13

**Authors:** Jeeraphong Thanongsaksrikul, Wanpen Chaicumpa

**Affiliations:** Laboratory for Research and Technology Development, Department of Parasitology, Faculty of Medicine Siriraj Hospital, Mahidol University, 2 Prannok Road, Bangkok-noi, Bangkok 10700, Thailand; Email: gskmu@hotmail.com

**Keywords:** botulinum neurotoxin, botulism, zinc metalloprotease, immunotherapy, serum therapy, therapeutic antibody, chimeric antibody, humanized antibody, single chain antibody variable fragment (ScFv), heavy chain antibody (HCAb), single domain antibody (sdAb), VH, VL, V_H_H, humanized-camel phage display library, nanobody, transbody, cell penetrating peptide (CPP), phage display

## Abstract

Specific treatment is not available for human botulism. Current remedial mainstay is the passive administration of polyclonal antibody to botulinum neurotoxin (BoNT) derived from heterologous species (immunized animal or mouse hybridoma) together with supportive and symptomatic management. The antibody works extracellularly, probably by blocking the binding of receptor binding (R) domain to the neuronal receptors; thus inhibiting cellular entry of the holo-BoNT. The antibody cannot neutralize the intracellular toxin. Moreover, a conventional antibody with relatively large molecular size (150 kDa) is not accessible to the enzymatic groove and, thus, cannot directly inhibit the BoNT zinc metalloprotease activity*.* Recently, a 15–20 kDa single domain antibody (V_H_H) that binds specifically to light chain of BoNT serotype A was produced from a humanized-camel VH/V_H_H phage display library. The V_H_H has high sequence homology (>80%) to the human VH and could block the enzymatic activity of the BoNT. Molecular docking revealed not only the interface binding between the V_H_H and the toxin but also an insertion of the V_H_H CDR3 into the toxin enzymatic pocket. It is envisaged that, by molecular linking the V_H_H to a cell penetrating peptide (CPP), the CPP-V_H_H fusion protein would be able to traverse the hydrophobic cell membrane into the cytoplasm and inhibit the intracellular BoNT. This presents a novel and safe immunotherapeutic strategy for botulism by using a cell penetrating, humanized-single domain antibody that inhibits the BoNT by means of a direct blockade of the groove of the menace enzyme.

## 1. Introduction

Botulism is a severe paralytic illness caused by intoxication with botulinum neurotoxins (BoNT) produced by anaerobic bacteria of the genus *Clostridium*,* Clostridium botulinum* [1,2,3]. BoNT is one of the most toxic substances for humans [4]. From primate experiments, the toxin has an extremely low median lethal dose (LD_50_), *i.e.*, 1 ng per kg body weight [5]. BoNT is transmitted easily by aerosol [5]. Accordingly, the toxin has been attempted by terrorists to use as a biological weapon. BoNT has been designated by the Centers for Disease Control and Prevention (CDC), USA as a category A biological weapon, which is a similar to anthrax in its threat to humans [4,5].

### 1.1. Botulinum Neurotoxins (BoNTs)

There are seven BoNT serotypes designated A–G serotypes (BoNT/A-BoNT/G). *C. botulinum*, a spore forming, rod shape anaerobic bacterium produces mainly BoNT/A, BoNT/B, BoNT/C, BoNT/D, BoNT/E, and BoNT/F. BoNT/G is solely produced by *C. argentinense*; *C. butyrium *produces BoNT/E, and *C. baratii* produces BoNT/F [1,3]. Among the seven serotypes, BoNT/A is the most potent for humans [2]. Naturally, BoNT is associated to other bacterial proteins, *i.e.*, hemagglutinin (HA) and non-toxic, non-hemagglutinin (NTNH) protein in a form of BoNT-HA-NTNH complex [3]. Although functions of the clostridial HA and the NTNH proteins are still unknown, it is believed that forming a complex with these proteins not only renders the BoNT more resistant to the host gastric acidity, but also facilitates the toxin entry into the blood circulation by undefined mechanism [6,7].

### 1.2. Synthesis and Molecular Structure of BoNTs

BoNT are encoded by *bont* genes (~3880 bp) which are present on various genetic elements, depending on the species and strains of BoNT-producing clostridia [7]. The *bont *genes that code for BoNT/A, BoNT/B, BoNT/E and BoNT/F (*bont/A*, some *bont/B*, *bont/E* and *bont/F*) are located on the bacterial chromosome [7,8,9]; *bont/C *and *bont/D* are derived from bacteriophages [10,11]; and the *bont/G *and some *bont/B* genes are present on plasmids [12,13]. Sequence similarity of the *bont* genes coding for the seven BoNT serotypes ranged from 34% to 97% [7].

**Figure 1:**
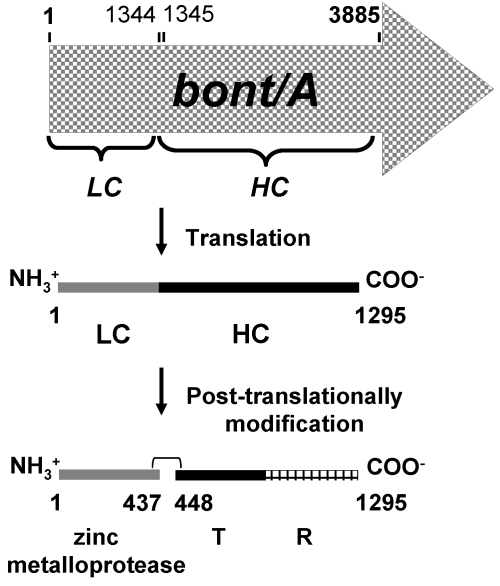
Schematic diagram of BoNT/A synthesis. Gene coding for BoNT/A (*bont/A*) synthesized a single polypeptide which is then nicked to form a di-chain active BoNT/A. LC, BoNT/A light chain (residues 1–437); HC, BoNT/A heavy chain (residues 448–1295); T, translcation sub-domain (residues 448–872); R, receptor binding sub-domain (residues 873–1295). A di-sulfide bond is formed by cysteine residues 430 and 454 [18].

The molecular structure of BoNTs has been revealed by crystallography as an A-B toxin [14,15]. It is believed that the two polypeptides are synthesized as a single polypeptide which is modified post-translationally by bacterial or host proteases to a 150 kDa, active di-chain holotoxin. Each molecule of the toxin is composed of an A subunit or light chain (LC, size ~50 kDa) which is linked to a B subunit or heavy chain (HC, size ~100 kDa) by a single disulfide bond. HC composed of two polypeptide sub-domains, *i.e.*, receptor binding (R) and translocation (T). LC of the BoNT is endowed with zinc-dependent metalloprotease activity [16]. Schematic diagram of BoNT/A synthesis is illustrated in Figure 1. The sequence variations of BoNT are in the R domain and the substrate interaction sites of LC domain [14,17]. 

### 1.3. Mechanism of Action of BoNTs

Ingested BoNT absorbed from the intestine, enters lymphatics which are later carried by blood circulation and distributed throughout the host tissues [19]. At a peripheral nerve ending, the R sub-domain binds to two receptors of the neuron, *i.e.*, low-affinity gangliosides and high-affinity synaptic vesicular proteins [20,21]. The synaptic vesicular proteins that serve as the BoNT receptors are normally located within the lumen of acetylcholine-containing vesicles but become exposed after the neurotransmitter release. Synaptic vesicular proteins that have been identified as BoNT/A and BoNT/B receptors are synaptic vesicular protein-2 (SV2) [22] and synaptotagmin-II (SytII), respectively [20,21]. The BoNT that has bound to the neuronal surface receptors then enters the cell *via* the receptor-mediated endocytosis (RME). Acidic pH of the endosome facilitates structural change of the T sub-domain, which forms a putative pore-like structure [23,24]. The partially or completely unfolded LC translocates across the endosomal membrane via the T-forming pore into the cytoplasm [24,25]. The free LC then refolds and specifically cleaves one of soluble *N*-ethylmaleimide-sensitive factor-attachment protein receptor (SNARE) proteins including synaptobrevin (or vesicle associated membrane protein; VAMP) which anchors on outer shell of synapticular vesicle, synaptosomal-associated protein of 25 kDa (SNAP25), and syntaxin. The latter two are associated with the inner leaflet of the neuronal plasma membrane [26]. BoNT/A, BoNT/C and BoNT/E cleave SNAP25 at different peptidase sites [27,28,29]. BoNT/C also digests syntaxin [25,30,31]. BoNT/B, BoNT/D, BoNT/F, and BoNT/G cleave synaptobrevin [7]. Table 1 gives details on the target SNARE proteins of the BoNTs and their specific endopeptidase cleavage sites. The cleavage of SNARE proteins, which are neurotransmitter exocytosis machinery, leads to abolition of the membrane fusion between neurotransmitter-containing vesicles and the neuronal plasma membrane, and eventually blocks acetylcholine release to neuromuscular junction, resulting in weakness of muscles or flaccid paralysis; the disease which is called botulism [5,26].

**Table 1:** Cleavage sites of different BoNTs on the human SNARE proteins [18].

BoNT Serotype	SNARE Substrate(s)	Susceptible Scissile Bond	Reference(s)
A	SNAP25	Gln197-Arg198	[27,28,29]
C	SNAP25	Arg198-Ala199	[30,31]
	Syntaxin	Lys253-Ala254 and Lys260-Ala261	[25,30,31]
E	SNAP25	Arg180-Ile181	[28,29]
B	VAMP	Gln76-Phe77	[32]
D	VAMP	Lys59-Leu60	[28,34]
F	VAMP	Gln58-Lys59	[33,34]
G	VAMP	Ala81-Ala82	[35]

### 1.4. Botulism

Botulism is the weakness of the affected striated muscles (flaccid paralysis) due to the BoNT mediated blockade of the release of acetylcholine neurotransmitter from pre-synaptic neuron into the neuromuscular junction [26]. Human botulism is caused by BoNT/A, BoNT/B, BoNT/E, and occasionally BoNT/F. Domesticated animals including dogs, cattle, birds, and chickens are affected by BoNT/C. BoNT/D can cause the disease in cattle. There has been no reported case of human or animal botulism associated with BoNT/G [3,5,36].

Four clinical types of human botulism have been identified. They are: food-borne-, infant-, wound- and inhalational botulism [5]. Food-borne botulism is manifested after ingesting improperly cooked, preserved or stored foods, especially canned food containing preformed BoNT. Under anaerobic conditions, bacterial spores that have contaminated the food germinate and release the BoNT by a cell-wall exfoliation mechanism. Unlike food-borne botulism, infant botulism is caused by BoNT released from growing clostridia that colonize in infant’s intestine [1,5]. In wound botulism, clostridia colonizes an infected injured tissue, produce toxins which later absorbed into the bloodstream of the host, and subsequently reach the neuromuscular junctions [1,5]. The inhalational form of botulism is man-made and caused by inhalation of the BoNT powder, which is aerosolized and transmitted to victims for the purpose of bioterrorism [5]. Among the four types, food-borne botulism is the most common type [1,5]. Clinical manifestations of the food-borne botulism usually begin 12 to 48 h after ingesting the food contaminated with the preformed BoNT [5,7]. 

### 1.5. Clinical Manifestations of Human Botulism [5,36]

All forms of human botulism present mostly identical neurological signs because all BoNT mediate similar physiological abnormalities in the peripheral nervous system. However, the neurological signs in food-borne botulism may include abdominal cramps, nausea, and vomiting. The neurological form is characterized by symmetrical, descending, and flaccid paralysis of parasympathetic nerves. The symptoms usually begin with cranial nerve (face) palsies, including drooping of the upper eyelids (ptosis), double vision (diplopia), blurred vision, difficulty in articulating words (dysarthria), difficulty in speaking (dysphonia), and difficulty in swallowing (dysphagia). The paralysis then develops to general weakness of several muscles such as arms, legs, and diaphragm which may lead to fatality if not treated properly and promptly. The severity of the disease varies from individual to individual and depending upon the amount of toxins consumed and absorbed [5].

## 2. Treatment of Botulism

Presently, there is no licensed BoNT antagonist, although several attempts have been made to invent the toxin inhibitors. Mouse monoclonal antibodies to BoNT light chain were unable to neutralize the BoNT mediated lethality in mice, although the antibodies injected intracellularly into nerve ganglion prior to the BoNT administration were found to prevent the BoNT mediated inhibition of neurotransmitter exocytosis in *Aplysia* [37]. Small molecular inhibitors of S_1 _subsite of type B BoNT metalloprotease were shown to inhibit the BoNT activity *in vitro* [38,39]. However, due to their inability to cross plasma membrane, none of them have reached the clinical trial for the human therapeutic value. 

The treatment of botulism is based on supportive measures including artificial respiration and passive administration of human and animal (mainly horse) derived anti-BoNT immune globulin (polyclonal antibodies; PAb) to the afflicted individual [5]. Immune sera and antibody preparations that have been used for treatment of human botulism are listed in Table 2. 

**Table 2:** Various anti-BoNT preparations for current therapeutic use.

Preparation	Target BoNT Serotype	Enterpreneur	Status	Reference(s)
1. Trivalent equine antitoxin	A, B, and E	CDC/USA	FDA approved	[36,40]
2. Bivalent equine antitoxin (BAT-AB)	A and B	Sanofi Pasteur Limited	FDA approved	[41,42]
3. Human botulism immune globulin (BabyBIG or BIG-IV)	A,B,C,D, and E	California Department of Public Health	FDA approved	[41,42,43,44]
4. Monovalent equine antitoxin (BAT-E)	E	Sanofi Pasteur Limited	Investigational	[41]
5. Heptavalent equine antitoxin (HBAT)	A to G	Cangene Corporation	Investigational	[41]
6. Recombinant antitoxin	A	University of California, San Francisco	Investigational	[36,45,46,47]

There are some limitations in using the immunized human/animal derived antitoxin. It is believed that the PAb derived from the immune serum can only block the circulating BoNT from cellular entry by inhibiting the binding of the toxin to the neuronal cell surface receptors [48,49]. They cannot traverse the plasma membrane. The preparation usually contains a relatively small proportion of specific immunoglobulins. Moreover, the animal-derived anti-BoNT is foreign to the human immune system. Therefore, not only are the antibodies speedily eliminated from the recipient’s body (hence large amount of the therapeutic antibodies are required to resuscitate the patient), but also the animal proteins elicit anti-animal isotypic response leading to adverse reactions which may be as serious as acute anaphylaxis and/or serum sickness [5]. The latter is mediated by immune complexes formed in the condition of the antigen (animal protein) excess. Besides, the supply and availability of the antitoxin are relatively limited because a prolonged immunization process is required before a satisfactory level of the antitoxin is reached in the sera. The quality of the BoNT neutralizing antibodies obtained in this way is subjected to batch-to-batch variation. The human recipient is also at a risk of anthroponosis/zoonosis. Therefore, alternative approaches for producing the botulism therapeutic antibody should be sought.

## 3. Therapeutic Antibody

### 3.1. Serum Therapy

Antibody has been used since the early nineteenth century in passive immunization for disease prevention and intervention as well as for therapeutic purposes [50]. The medical practice was then called “Serum therapy”. The therapeutic preparations were either immune serum or immunoglobulins derived from plasma or serum of healthy, vaccinated and/or disease convalescent humans. For example, the serum of a human immunized with tetanus toxoid was used for treatment of tetanus [51]; and the serum of a convalescing measles patient was used in the intervention of measles morbidity in an immunocompromised child who had been exposed to the measles virus [52]. The immunoglobulin/antibody therapeutic role include restoration of the immunoglobulin functions in the immunodeficient subjects, toxin/enzyme neutralization, prevention of pathogen attachment to host cell/tissue and colonization, prevention of cellular entry, inhibition of motility, promotion of phagocytosis by means of opsonization followed by killing, immune exclusion, antibody dependent cell mediated cytotoxicity (ADCC) by natural killer (NK) cells and antibody-complement mediated lysis of pathogens or infected host cells [53,54,55,56,57,58]. Limitations in using human derived antibody include the scarcity of donors, variation in antibody quality, and recipient risk to some blood-borne anthroponosis. The practice is also unethical. Thus, therapeutic antibodies were from immunized animals such as horse or sheep. Nevertheless, there are still problems in production and use of the animal-derived antibody. Repetitive immunizing doses and a prolonged immunization process (more than 6 months or a year) are required before a satisfactory level of antibody is obtained from the plasma/serum of the immunized animal. There is a batch-to-batch variation in the antibody quality. Using *in vivo* immunization, it is difficult to produce immune serum for low immunogenic and/or highly toxic molecules (such as snake neurotoxin), for which the immunogenic dose is much higher than the toxic/lethal dose, similarly for small molecular hapten that contain only B cell epitope, such as puffer fish tetrodotoxin (~320 Da). Besides, large animals require a large amount of space and care. Animal immune serum contains a large proportion of non-specific serum proteins/immunoglobulins. Most of all, animal proteins are foreign and highly immunogenic to the human immune system, often leading to allergic reactions such as anaphylaxis and serum sickness—the latter is caused by human anti-animal isotypic antibodies which form an immune complex with the animal proteins. The recipient is also at risk of zoonosis. 

### 3.2. Mouse Monoclonal Antibody

The invention of hybridoma technology by Köhler and Milstein (subsequent recipients of Nobel Laureates) in 1975 [59] has abrogated many limitations in preparing and using the animal immune serum. The mouse monoclonal antibody (MAb) with high purity, defined specificity, and reproducible affinity [60] can be obtained easily, rapidly, and adequately without frequent/repeated and prolonged immunization process but merely by growing an established hybridoma clone in a culture medium. The recipients of the MAb are relatively safe from zoonosis as the MAb can be obtained from the spent culture medium of a hybridoma grown in a serum free medium. Nowadays, murine MAb have been used as therapeutic agents against cancers, autoimmune, inflammatory conditions, infectious diseases, and intoxications [53,61]. Currently, about 30 therapeutic antibodies have been approved by FDA for marketing in the United States and over 200 MAb candidates are in the pipeline [62,63]. Recently, MAb specific to HC of BoNT subtype A1 were produced [64]. The MAb and its pegylated F(ab)’_2_ not only neutralized the BoNT activity but could rescue the intoxicated mice from the toxin mediated lethality [64]. Nevertheless, limitations in production of MAb include requirement of tissue culture facility. Often the culture is contaminated and the hybrid clone loses the immunoglobulin genes [65]. Biologically, the mouse MAb is foreign to the human immune system and the human antibody to mouse antibody (HAMA) is produced inevitably which also leads to adverse effects [66,67,68]. Ideally, an antibody with minimal- or, to the best, no immunogenicity to the human immune system is needed for human therapy. 

### 3.3. Chimeric-, Humanized- and Single Chain Antibodies

The first attempt to reduce immunogenicity of the mouse MAb in the human recipient was to produce a human-mouse chimeric antibody [69]. The Fc portion of the mouse antibody was replaced by the human counterpart in the chimeric molecule by genetic engineering. The Fc is required to retain effective therapeutic mechanisms (Section 3.1) and longevity as well as for clearing the immune complexes by means of Fc-dependent phagocytosis. The chimeric antibody retains the original antigenic (epitopic) specificity of the mouse MAb but about 70% or more of the molecule is human protein [70]. F(ab)’_2_, Fab or single chain antibody fragments (ScFv; VH linked to VL by a short polypeptide) can be used in human therapy when the bio-function of the Fc in mediating the therapeutic effect is not needed. Clearance of the immune complexes formed by these antibody fragments can be mediated by Fc-independent phagocytosis [71,72]. The immune aggregates and small molecules (e.g., free ScFv) may be filtered through glomeruli for which the filtration cut-off is approximately 60 kDa [73]. However, HAMA is still produced against the mouse antibody fragments [66,67]. Further reduction of the immunogenicity of the mouse protein in the human recipient has been done by replacing the mouse immunoglobulin frameworks (FRs) with the best matched human counterparts. This can be done by the molecular grafting of all of the mouse complementary determining region (CDR) coding sequences onto the DNA sequences of human FRs; the process is called “Humanization” [74,75]. The humanized-mouse monoclonal antibody fragments such as ScFv retain the original epitopic specificity, but the mouse protein is contained only in the CDRs and is unlikely to be immunogenic in humans. The first chimeric (antithrombolism) and humanized (immunosuppressant) antibodies for human use were approved by the FDA in 1994 and 1997, respectively [76]. For anti-BoNT, several chimeric, humanized and ScFv are currently in pre-clinical evaluation [45,46,47]. 

### 3.4. Fully Human Monoclonal Antibody

The production of fully human therapeutic monoclonal antibody is possible nowadays by using several approaches. Transgenic mice carrying human immunoglobulin genes can be immunized with an antigen. The animals produce human polyclonal antibodies which are the products of the human immunoglobulin transgenes [77]. However, the supply of the human polyclonal antibodies from the transgenic mice is limited and expensive and thus they are limited to research purposes. Spleen cells of the immunized transgenic mice can be fused with mouse myeloma cells, and hybridomas secreting human monoclonal antibodies can be produced by conventional hybridoma technology. However, the transgenic facility and the cost of transgenic mice are hardly affordable by most developing countries. The alternative production of a fully human monoclonal antibody is done by infecting human immune B lymphocytes from immunized/disease convalescing subjects with Epstein-Barr (EB) virus [78,79,80]. The virus transforms the B cells into immortalized cells which can be grown individually in tissue culture for human monoclonal antibody production. Nevertheless, the EB transformed B cells frequently lose the ability to secrete the MAb [81]. 

Theoretically, the human immune system can generate approximately 10^12^ of antibody diversity from a rather limited immunoglobulin gene repertoire by means of several notated mechanisms including: the use of multiple germ-line gene segments, combinatorial V-(D)-J joining, junctional flexibility of coding joints, P-region nucleotide addition, *N*-region nucleotide insertion for H chains, combinatorial association of L and H chains, and somatic hypermutation and affinity maturation in secondary lymphoid tissues [82,83]. 

Contemporary technology in molecular biology and molecular immunology such as antibody engineering also allows the possibility of making a specific human antibody with relatively high affinity to a target molecule *in vitro* (without the *in vivo* immunization). For instance, the human immunoglobulin gene repertoire could be generated *in vitro* by cloning the antibody coding genes from B lymphocytes of naïve or specifically immunized donors into a display system, such as cell [84,85,86], ribosome [87], and phage [88] to construct the so-called “Human antibody display library” [89,90]. Among these display systems, phage is the most commonly used for constructing the antibody display library [89]. The antibody molecules; mostly in the fragment formats (Fab, ScFv, single antibody domain) [91], are displayed on the surface of fd or M13 filamentous bacteriophages which the phenotype of each displayed molecule is correlated to the genotype of the encoding DNA in the phage genome [92]. To construct such a recombinant antibody phage display library, a pool of differently rearranged immunoglobulin genes is amplified and cloned into a phagemid vector (a plasmid containing a phage origin of replication). The translation frame of the gene is positioned next to gIII or gVIII, which code for phage pIII minor coat protein and pVIII major coat protein respectively [93,94]. There is an amber stop codon (TAG) incorporated between the inserted antibody gene and gIII or gVIII [93]. In the suppressor *E. coli* strain, the suppressor tRNA is produced, which allows reading through the amber codon so that the antibody is displayed on the phage surface by fusing with the pIII or pVIII protein [93]. In contrast, non-suppressor *E. coli* strain does not produce such a tRNA; thus the recombinant antibody is secreted alone without the pIII or pVIII phage partner [93]. The recombinant phagemid-carrying antibody gene insert will replicate in the suppressor *E. coli*. Mature phage (complete) phage particles are obtained after co-infecting the cultured phagemid transformed *E. coli *with a helper phage such as M13K07, so called “Phage rescue” process. The newly assembled recombinant phage particles display the antibody molecules on their surface as pIII- or pVIII-fusion protein. The co-infection of the recombinant phagemid transformed *E. coli* with the helper phage is indispensible because the phagemid genome only encodes the pIII or pVIII fusion proteins without other genes for all other essential structural phage proteins; thus the mature phage particles cannot be produced. The phage structural proteins must be provided by the helper phages. The helper phages also help in packaging the replicating recombinant phagemids into the capsid. After phage rescuing, some new helper phages could be obtained in the phage library [93]. Nevertheless, they are less competitive for assembling into complete particles than the recombinant phagemids because of their defect in the origin of replication [93].

A phage that displays an antibody to a particular antigen can be directly isolated from the constructed antibody phage display library by means of “Bio-panning”. An antigen is immobilized on a solid phase, such as cell surface, beads, or wells of ELISA plate. The antigen binds with the displayed-antibody molecule on the phage particle. The antigen-unbound phages are removed simply by extensive washing with a buffer such as phosphate buffered saline (PBS) containing a mild detergent, e.g., Tween-20 (PBST). The antigen-bound phages can be eluted, further amplified in the suppressor *E. coli* and rescued from the culture after adding the helper phage. They can be subjected to repeated bio-panning rounds in order to enrich the high affinity binders [93]. Alternatively, the antigen-bound phages from the single bio-panning round can be added directly with a non-suppressor strain of *E. coli* host cells to allow phage transfection into the bacteria. In our laboratory, this “Single round bio-panning” has been used successfully for the selection of recombinant phage clones that display specific antibodies to a variety of target molecules [90,95,96,97]. In the *E. coli* transfection, each recombinant phage uses the unoccupied pIII molecule(s) to bind to the bacterial F pilus. The latter serves as a molecular tube through which the phagemid genome with the antibody gene insert enters the bacterial cytoplasm. The phage-transfected *E. coli* colonies grow on a selective agar plate and can be selected appropriately [90,95,96,97]. The selected transformed non-suppressor *E. coli* can be grown under an induction condition (usually by isopropyl β-D-1-thiogalactopyranoside; IPTG) to express the soluble recombinant antibody molecules with fusion tags such as E-tag. The tagged recombinant antibody can be detected and purified easily by using anti-tag monoclonal antibody as the capture reagent. The bacterial expression system allows a production of the fully human recombinant antibody at the desired quantity without the *in vivo* immunization [93,98]. In addition, the antibody coding gene can be subcloned from the phagemid into other appropriate vectors for antibody production by other cellular factories such as insect, yeast and mammalian. The antibody display technology allows also the production of antibodies to low immunogenic, non-immunogenic and/or toxic molecules which are highly difficult, if possible, by a conventional immunization method. The *in vitro* production is free from an *in vivo* immune regulation and feedback mechanism. The antibody recipient is safe from zoonosis. Moreover, the antibody production is free from the animal ethic and welfare concern. 

### 3.5. Single Domain Antibody (sdAb)

Serum of some camelids, *i.e.*, *Camelus dromedarius* (one hump Arabian camel), llama (*Lama glama* and* L. guanicoe*) and alpaca (*Vicugna pacos*) contains both a conventional four-chain antibody and an atypical antibody, called “Heavy-chain antibody (HCAb)” the molecule of which does not contain a light chain. Instead, two heavy chain molecules tend to be associated by non-covalent bonding [99]. HCAb has one antigen-binding site per molecule and this is contributed by the heavy chain variable domain designated “V_H_H” instead of the typical VH and VL of the conventional four-chain antibody. Because the HCAb lacks VL, the hydrophobic amino acids V42, G49, L50, and W52 of the conventional VH where the VL partner used to associate were replaced by hydrophilic counterparts, *i.e.*, F/Y42, E49, R/C50, and G/L52 in the FR2 of the V_H_H in order to reduce molecular aggregation and increase serum solubility of the HCAb [100,101]. The amino acid tetrad is a hallmark that allows distinction to be made between the V_H_H of HCAb and the conventional VH. 

Diversity of the HCAb repertoire is generated from the germline immunoglobulin DNA by the same mechanisms as for humans and mice, except that the combinatorial H-L chain pairing is absent because of the lack of the L chain partners [102,103]. The HCAb diversity was from: multiple germ-line gene segments, combinatorial V-D-J joining, junctional flexibility, P-nucleotide addition and N-nucleotide insertion. Somatic hypermutation and affinity maturation also occur in the secondary lymphoid tissues [103]. The camelid immune system compensates for the absence of combinatorial H-L chain pairing by using several other strategies which have been described in more detail elsewhere [103]. Briefly, the HCAb V_H_H segments have expanded surface for antigen-contact especially in the CDR1 and CDR3 [102]. CDRs of the V_H_H are unusually longer than the CDRs of the conventional VH; for example, CDR1 and CDR3 of VH have 5 and 12 amino acids respectively, while V_H_H CDR1 and CDR3 have 8 and 16–18 amino acids respectively [103,104]. It is believed that the longer CDR3 of camel V_H_H is facilitated by an increased activity of the TdT enzyme (*N*-nucleotide insertion) during the V-D-J joining [105]. Besides, there are several hotspots for somatic mutations by the oligonucleotide insertions and deletions in V_H_H DNA which are frequently found at CDR1 and CDR2 [102,105]. Moreover, the camel V_H_H segments have structural hypervariability of the antigen binding loops created by non-canonical extra-loop disulfide bonds in the CDRs, including CDR3 intra-loop disulfide bonds and the inter-loop disulfide bonds that link the CDR3 with CDR1 or CDR2 and with FR2 [100,106].

## 4. Molecular Insertion of a Single Domain Antibody (sdAb) into the Enzymatic Groove Directly Inhibits BoNT Zinc Metalloprotease Activity: A Novel and Specific Immunotherapeutic Approach to Botulism

CDR3 of V_H_H has been shown to be penetrable into an active-site pocket of a target enzyme which would never be reached by a large, conventional antibody [106,107]. Consequently, the camelid V_H_H antibody fragments have been shown to be potent enzyme inhibitors [108,109]. Because of its characteristics [small size (~15–20 kDa), high tissue-penetrating ability, high yield from an expressed system such as *E. coli* (5–10 mg per mL), and high stability], the single domain antibody (sdAb) has drawn much attention regarding its use as a therapeutic antibody in human cancers, infectious diseases, and intoxications, particularly those caused by toxic enzyme molecules [97,110,111] or toxic small molecules such as puffer fish tetrodotoxin [112]. The camel V_H_H (Nanobody^®^, Ablynx) specific to von Willebrand factor has been found to be safe in human subjects (no adverse reaction) in the phase I clinical trial [62]. The safety should be due to the high degree of sequence homology between human VH and camel V_H_H immunoglobulin frameworks (FRs) which is >80% [111]. The small size of the antibody is necessary not only for high tissue penetrating ability but also in having a more defined antigen binding site than the Fv of the conventional antibody or recombinant ScFv [113]. 

Recently, we have constructed a humanized-camel VH/V_H_H phage display library [97]. Complementary DNA prepared from mRNA of a *Camelus dromedrarius* was used as a template for PCR amplification of the gene sequences coded for camel VH/V_H_H. However, primers used in the PCR amplification were degenerate primers designed from all families of human VH and JH sequences including VH 1a, 1b, 1c, 1d, 2a, 2b, 3a, 3b, 3c, 4a, 4b, 5a, 6a and 7a and JH3, J6 and J1245 [90]. The human primers created an amplification constraint during the polymerase chain reaction which only the human-like camel VH/V_H_H (humanized) sequences were amplified [97]. V_H_H that bound specifically and neutralized the enzymatic activity to BoNT/A light chain was produced from a non-suppressor *E. coli* clone carrying recombinant V_H_H-phagemid that had been selected from the humanized-camel VH/V_H_H display library. The epitope of the V_H_H was found to be a peptide located at the immediate vicinity of the BoNT/A enzymatic active site. Molecular docking and interface binding revealed that the V_H_H inserted its CDR3 into the toxic metalloprotease cleft [97]. The V_H_H has high sequence homology to the human VH sequences [97]. Therefore, the single domain anti-BoNT/A antibody should have high potential as a safe, as well as effective and specific therapeutic remedy for human botulism caused by the type A BoNT. Recently, V_H_H specific to toxoid of BoNT/A was also produced from a phage display library constructed from B cells of a non-immune llama [114] as well as a llama immunized with the BoNT toxoid [115]. It is known, however, that there are sequence variations of the BoNT catalytic domain within the areas near to the enzymatic groove, and these variations have contributed to substrate specificity of different BoNT subtypes. Thus the humanized V_H_H that blocks the enzymatic activity of BoNT/A subtype should not cross-neutralize the other BoNT subtypes. Nevertheless, the same strategy [97] can be applied for production of humanized-camel V_H_H that inhibit other BoNT subtypes. 

## 5. Cell Penetrating Antibody (Transbody)

It is known that the enzymatic active site of the BoNT light chain is only exposed after the light chain has been released into the cytoplasm of the affected neuronal cells [14]. Therefore, the BoNT enzymatic cleft is inaccessible by the extracellular antibody. This problem can be overcome by making the V_H_H into a cell-penetrating format or “Transbody”. For this procedure, V_H_H is linked molecularly to a suitable cell-penetrating peptide (CPP) [116,117,118]. CPP is a short peptide that can be conjugated to various cargo molecules including proteins, antibody, nucleic acids, plasmid, and siRNA [119]. The CPP directs its cargo (in the form of a chimeric molecule) into the cytosol without causing cellular damage [118] by as yet unknown mechanism(s) [120,121,122,123,124]. CPPs have become a promising vehicle for cellular uptake of therapeutic molecules [125]. In addition, CPP sequences can be modified for delivering the cargoes to different subcellular compartments [126]. Various CPPs and their amino acid sequences have been listed elsewhere [127]. Recently, a *cpp*-plasmid platform has been established in our laboratory [118]. The coding sequence of the V_H_H that has been shown to inhibit the BoNT enzymatic activity can be molecularly linked to a CPP coding sequence using the plasmid platform. The CPP-V_H_H fusion protein is expected not only to traverse across the neuronal cell membrane into the cell but also to retain the BoNT inhibitory activity which would block the intracellular enzymatic target. This approach has high potential as a novel and specific immunotherapeutic strategy for human botulism. 

## 6. Conclusions

Treatment of human botulism is supportive and symptomatic. Passively administered animal-derived anti-BoNT neutralizes circulating botulinum neutrotoxin by inhibiting binding of the toxin to neuronal receptors. The antibody cannot inhibit directly the proteolytic activity of the toxin that has gained cellular entry. In order to be mostly effective the anti-BoNT must be given at the earliest phase of the illness. The supply of the antitoxin is limited and not always available. Moreover, the animal protein induces adverse reactions in the recipient. A humanized single domain monoclonal transbody (cell penetrating V_H_H) that binds and blocks the zinc metalloprotease activity of the intracellular BoNT specifically and directly, and does not cause the undesirable side effects, should be a novel and better immunotherapeutic remedy for human botulism. 
